# A case of severe acute hemorrhagic duodenitis after administration of immune checkpoint inhibitor

**DOI:** 10.1002/deo2.19

**Published:** 2021-08-22

**Authors:** Keita Saito, Hironobu Nagumo, Miyuki Iwasaki, Takuro Nishiwaki, Daiki Ozono, Shin Inoue, Souhei Yoshimura, Hideyuki Kishita, Kenichiro Nakachi, Hisato Harasawa, Natsuki Kawamitsu, Shigenobu Yoshimura, Toshiyasu Shiratori, So Nakaji, Hiroyuki Ito

**Affiliations:** ^1^ Department of Gastroenterology Kameda General Hospital Chiba Japan; ^2^ Department of Respiratory Medicine Kameda General Hospital Chiba Japan

**Keywords:** atezolizumab, immune checkpoint inhibitor, immune‐related adverse events

## Abstract

*Case*: A 66‐year‐old man started carboplatin + etoposide + atezolizumab therapy for advanced small cell lung cancer. Seventeen days after the start of treatment, the patient presented with hematemesis and underwent emergency endoscopy, which revealed multiple erosions and ulcers in the duodenum. Some ulcers showed pulsating bleeding, which was stopped by clipping and cauterization using hemostats. Biopsy of the mucosal peri‐ulcer showed lymphocyte, eosinophil, and plasma cell infiltration. The patient was suggested to have acute hemorrhagic duodenitis, which was associated with immune checkpoint inhibitors (ICIs), and conservative treatment with blood transfusion and antacids was continued. However, 11 days after hemostasis, bleeding from a new ulcer was observed. Hemostasis was achieved by coagulation and clipping again, but the general condition of the patient deteriorated owing to the rapid progression of the primary disease, and he died 8 weeks after the start of treatment.

*Discussion*: Although there have been several reports of colitis and other adverse events caused by ICIs, there have been very few reports of duodenitis. Endoscopic findings include diffuse erythema, erosions/ulcerations, and villous atrophy, and pathological findings include eosinophilic infiltration and increased levels of CD8‐positive T cells. However, there have been no reports of duodenal mucosal damage caused after administration of atezolizumab nor of severe cases of massive bleeding requiring endoscopic hemostasis and blood transfusion, as in this case.

## INTRODUCTION

Recently, immune checkpoint inhibitors (ICIs) have been used to treat various cancers. ICIs are monoclonal antibodies that target cytotoxic T‐lymphocyte antigen 4, programmed cell death 1 (PD‐1), and its ligand (PD‐L1), which are negative immunomodulatory molecules. Atezolizumab is a PD‐L1 antibody that has been shown to be effective in combination with carboplatin and etoposide for the treatment of advanced small cell lung cancer.^¹^ The side effects caused by ICIs are known as immune‐related adverse events (irAEs). However, there have been very few reports of duodenitis. To the best of our knowledge, there have been no reports of duodenitis caused after administration of atezolizumab and hemorrhagic duodenitis as an adverse event of any ICIs.

## CASE REPORT

**FIGURE 1:**
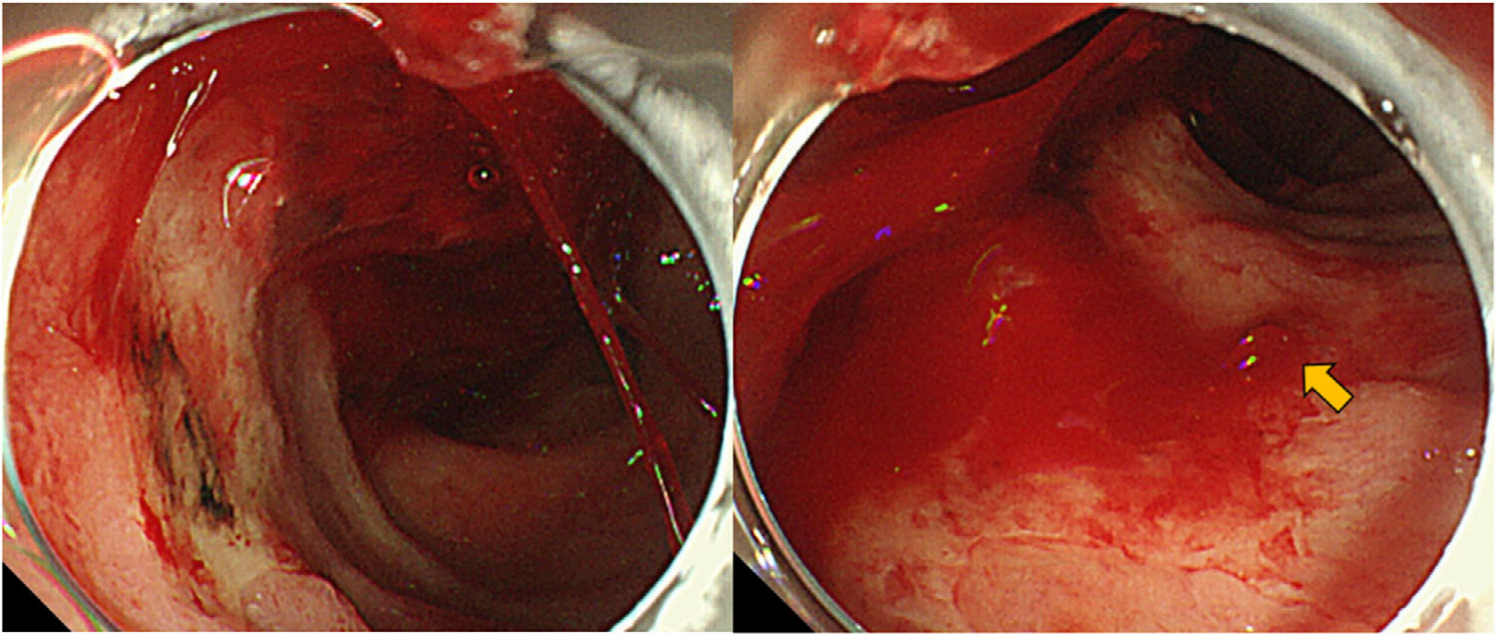
This is the duodenal finding of an emergency upper gastrointestinal endoscopy performed due to hematemesis. There were multiple erosions and ulcers in the duodenum. Pulsating bleeding was observed from an ulcer in the lower duodenal angle (arrow), and hemostasis was performed using hemostats and clips

**FIGURE 2:**
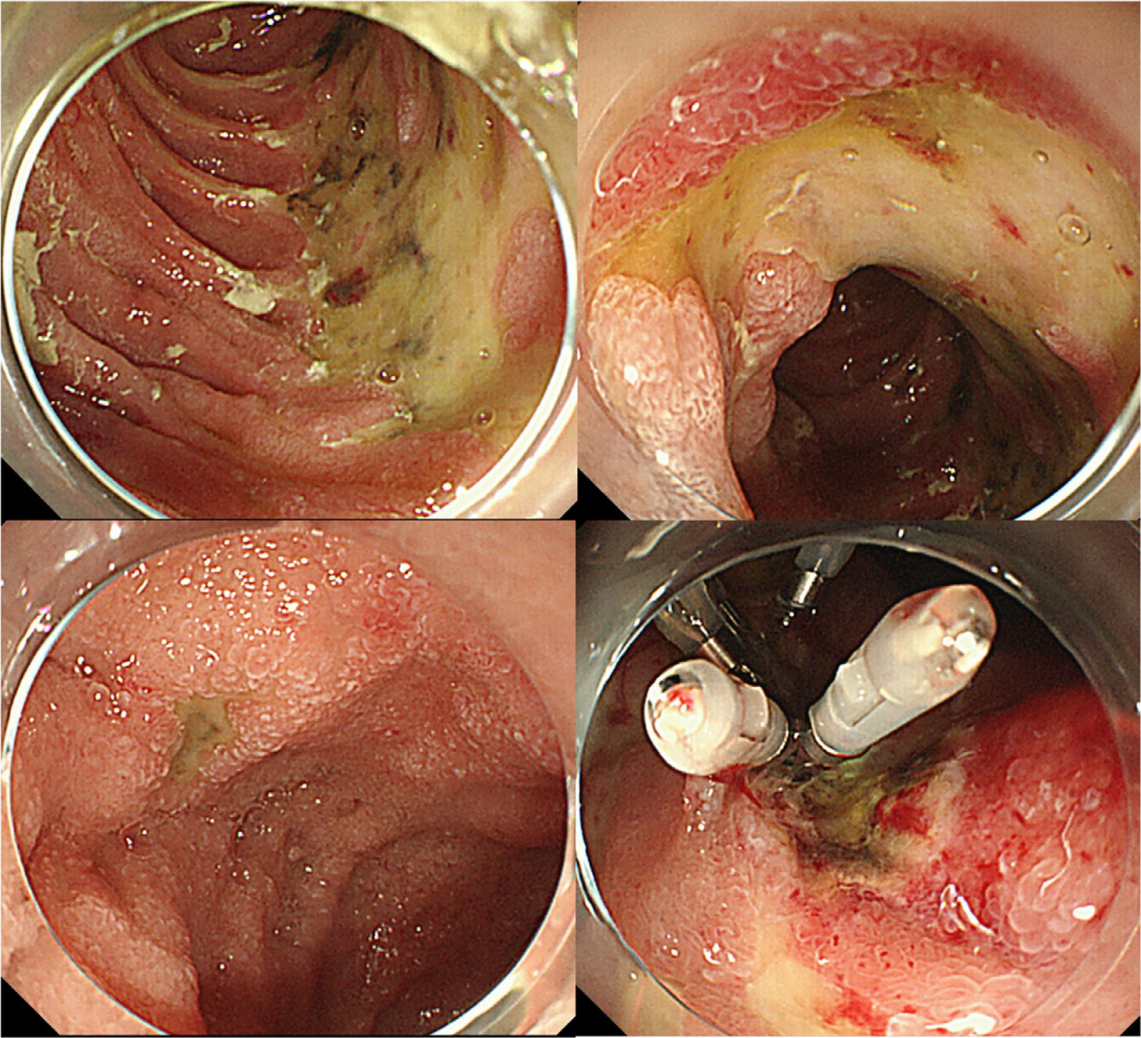
Multiple large and small erosions and ulcers were found in the duodenum. The clip used to stop the bleeding was still in place, and a tissue biopsy was performed from the nearby mucosa

**FIGURE 3:**
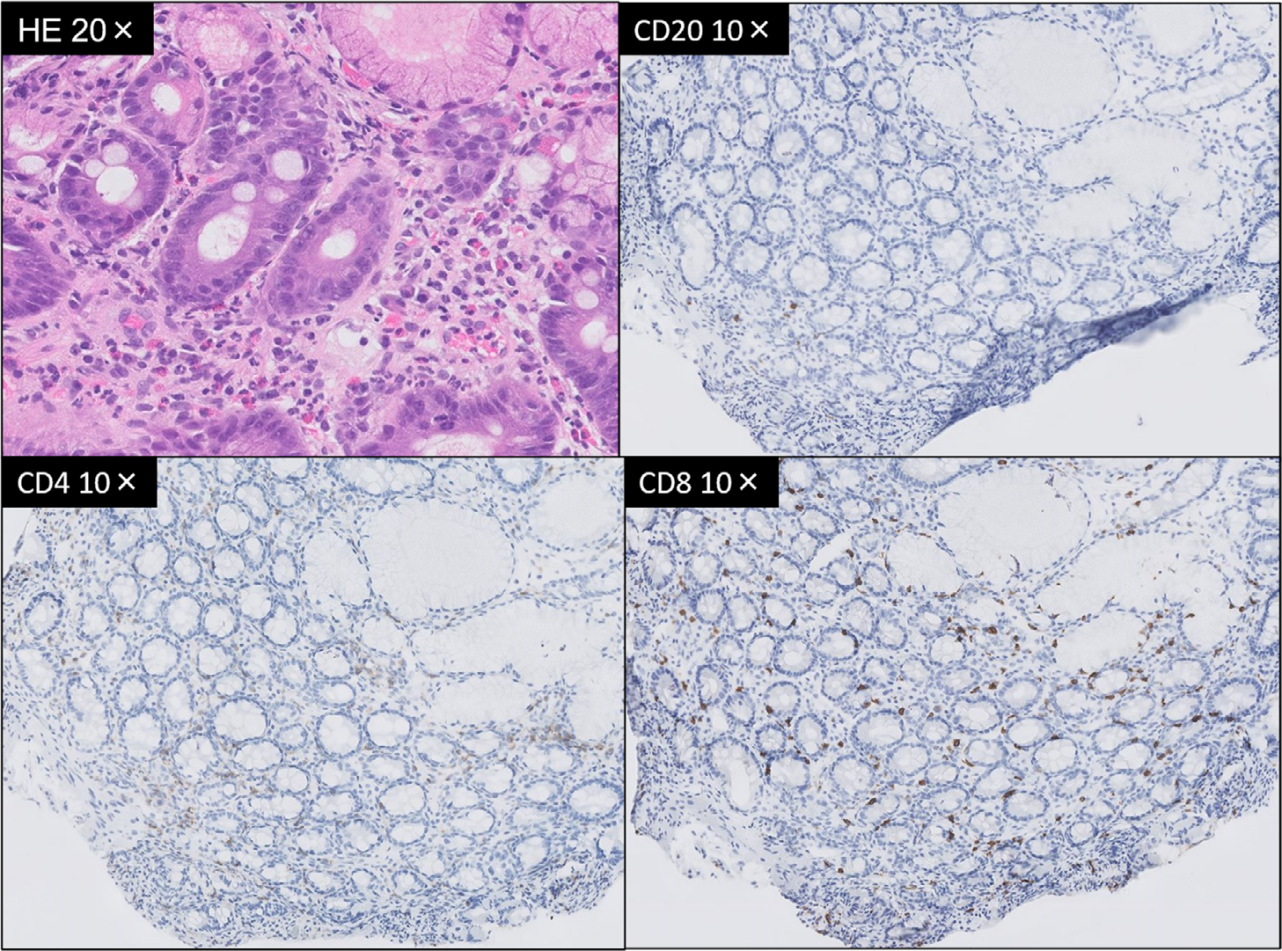
Hematoxylin‐eosin stained (HE) image of a duodenal biopsy (upper left). More than 20 eosinophilic infiltrates were conspicuous in one high power field. In immunohistochemistry, CD20‐positive cells were rarely seen. CD8 and CD4 were stained to the same degree, but CD8‐positive cells were increased when compared to the distribution of normal tissue

Carboplatin + etoposide + atezolizumab therapy was initiated in a 66‐year‐old man for advanced small cell lung cancer with multiple bone metastases at the department of respiratory medicine in our hospital. On the 10th day of treatment, he had vomiting and diarrhea; on the 15th day, computed tomography (CT) scan showed thickening of the duodenal wall, which was diagnosed as duodenitis. The patient was followed up with fasting and antacids. Although he was not taking any non‐steroidal anti‐inflammatory drugs or antithrombotic agents, he was referred to our department of gastroenterology because of hematemesis at night on the 18th day. On examination, he was in a state of shock, his face was pale, and had tenderness in the upper abdomen. Emergency endoscopy revealed multiple erosions and ulcers in the duodenum (Figure 1). As some ulcers showed pulsating bleeding, hemostasis by clipping and cauterization with hemostatic forceps were performed. On subsequent endoscopic observation, a tissue biopsy sample was taken from the peri‐ulcer mucosa of the duodenum (Figure 2). The duodenal pathology image showed infiltration of lymphocytes, eosinophils, and plasma cells (Figure 3). In immunohistochemistry, CD20‐positive cells are rarely seen. Compared with that of normal tissues, the distribution of CD8‐positive T cells was increased.

Based on the endoscopic and histological findings, the diagnosis was acute hemorrhagic duodenitis caused after administration of ICI, and conservative treatment with blood transfusion and antacid administration were continued. There were no subsequent symptoms of diarrhea or bloody stool. Although the deep colon could not be observed, colonoscopy showed no abnormal findings of the mucosa. Four days after hemostasis, endoscopy showed that the ulcer was healing, and the patient was able to resume eating. However, bleeding occurred again 11 days after hemostasis, and spurting bleeding was observed from the new ulcer. Hemostasis was performed by cauterization using hemostats and clipping; nonetheless, the patient's general condition deteriorated rapidly owing to progression of the primary disease. In the end, both the patient and his family requested only supportive care, and he passed away 8 weeks after the start of treatment.

## DISCUSSION

In terms of frequency of irAEs, gastrointestinal toxicity has been found to be the second most common irAE after skin toxicity.^²^ However, the exact frequency of duodenitis caused by ICIs is unknown because there have been only 10 previous reports.^³^ ^⁻^ ^⁹^

In previous reports, the male to female ratio was 7–3, median age was 59 years, and median time of onset was 9.5 weeks (Table 1). With the exception of the present case, 10 patients have shown improvement in symptoms. Nine patients were treated with steroids, two of whom required additional infliximab, and one had symptomatic improvement with additional vedolizumab. In one case, symptoms improved without steroid use. The endoscopic findings varied from mucosal erythema, erosions, and ulcers to villous atrophy. Pathological findings included eosinophilic infiltration, increased apoptotic bodies, and increased CD4/CD8‐positive T cells.

**TABLE 1:** Duodenitis caused by ICIs

Year	Author	Age	Sex	Primary disease	ICIs	Symptoms	Onset (weeks)	Endoscopic findings	Pathological findings	Treatment
2016	Messmer	83	M	Melanoma	Ipilimumab	Abdominal pain, Diarrhea	4	Small ulcer	Villous atrophy, Increased apoptotic bodies	Prednisolone
		55	M	Melanoma	Ipilimumab + nivolumab	Loss of appetite, Diarrhea	7	Erythema	Increased eosinophils	Steroid
2016	Gonzalez	29	M	Melanoma	Ipilimumab + PD‐1 inhibitor	Abdominal pain, Diarrhea	4	Erythema, Erosion	Increased eosinophils	Steroid
		57	F	Melanoma	PD‐1 inhibitor	Diarrhea	60	Erosion	Increased eosinophils	Steroid
		56	M	Lung cancer	Nivolumab	Diarrhea	3	Small ulcer	Increased eosinophils	Withdrawal
2016	Onuki	68	M	Squamous cell lung carcinoma	Pembrolizumab	Loss of appetite, Abdominal pain	20	Erythema, Ulcer	CD4 and CD8 positive lymphocytes infiltrate	Prednisolone
2019	Yang	68	M	Melanoma	Ipilimumab + nivolumab	Abdominal pain, Diarrhea	33	Normal	Extensive eosinophilic infiltrate	Prednisolone
2019	Rapisuwon	60	F	Melanoma	Ipilimumab + nivolumab	Unknown	12	Erythema, Erosion	Inflammatory cell infiltrate	Prednisolone, infliximab, vedolizumab
2019	Duval	58	M	Renal cancer	Nivolumab	Diarrhea	6	Normal	Villous atrophy, CD4 and CD8 positive lymphocytes infiltrate	Methylprednisolone
2019	Hayashi	55	F	Ovarian cancer	Avelumab	Diarrhea	17	Villous atrophy	Lymphocytic inflammation, Eosinophil/Neutrophil infiltration	Prednisolone, infliximab
Our case		66	M	Small cell lung carcinoma	Atezolizumab	Hematemesis	2	Multiple ulcers	Increased Eosinophils, CD4 and CD8 positive lymphocytes infiltrate	Conservative

Abbreviation: ICIs, immune checkpoint inhibitors.

In our case, symptoms of vomiting and diarrhea were observed on the 10th day of treatment, but they improved in just 1 day. Furthermore, there were no symptoms of fever or abdominal pain, and the patient was able to eat. Therefore, we did not consider the effects of viral or bacterial infection. However, we should have performed cytomegalovirus (CMV) antigen tests and immunostaining of tissues to suspect CMV infection at the time of treatment, because immunosuppression is often observed in patients receiving chemotherapy, especially ICIs.

*Helicobacter pylori* antibody in the blood was negative, denying any current *H. pylori* infection. Additionally, radiation was performed on the thoracic and lumbar spine owing to multiple bone metastases; however, the duodenum was not included in the irradiation field. Blood gastrin levels were not tested, but the patient did not complain of abdominal symptoms before the start of treatment.

The drugs used included carboplatin and etoposide. However, we did not consider them as causative agents because the CT scan showed that inflammation was confined to the duodenum, and there have been no reports of such inflammation with these drugs.

Duodenal biopsy showed eosinophilic infiltration in the duodenal mucosa and CD4‐ and CD8‐positive T cells in the mucosal lamina propria. In duodenitis caused by ICI, Irshaid et al reported a significant decrease in the CD4:CD8 ratio compared to normal tissue.^10^ In our case, there was an increase in the number of CD8‐positive cells compared to normal tissue, but there was no obvious decrease in the CD4/CD8 ratio.

Although we could not completely rule out viral infection, we suspected duodenitis caused by ICI. Therefore, prednisolone administration should have been considered, as reported in previous cases. Unfortunately, we did not have adequate experience with irAEs at that time, nor did we have enough time to collect and review previous reports of duodenitis. Moreover, the patient and his family requested only supportive care because of his deteriorating general condition caused by the progression of the primary disease.

This is the first report of duodenitis caused after administration of atezolizumab. Clinically, the effect of atezolizumab was suspected, and it was suggested that it might cause more severe duodenitis in a shorter time than other ICIs. We consider that experience with a larger number of cases is needed to show this association.

Although the frequency of serious side effects such as our case is thought to be very rare, if a duodenal ulcer is observed after administration of ICIs, administration of steroids should be considered after a thorough differential diagnosis of viral, drug, or radiological causes.

## CONFLICT OF INTEREST

Authors declare no conflict of interests for this article.

## ETHICS STATEMENT

The authors determined that approval from the hospital's ethics review committee was not necessary in this report because it involved only one case. Additionally, informed consent was obtained from the patient and his family for treatment and publication.

## FUNDING INFORMATION

None.

## References

[1] Horn L , Mansfield AS , Szczęsna A , *et al*. First‐line atezolizumab plus chemotherapy in extensive‐stage small‐cell lung cancer. N Engl J Med 2018; 379: 2220–9. 30280641 10.1056/NEJMoa1809064

[2] Michot JM , Bigenwald C , Champiat S , *et al*. Immune‐related adverse events with immune checkpoint blockade: A comprehensive review. Euro J Can 2016; 54: 139–48. 10.1016/j.ejca.2015.11.016 26765102

[3] Messmer M , Upreti S , Tarabishy Y , *et al*. Ipilimumab‐induced enteritis without colitis: A new challenge. Case Rep Oncol 2016; 9: 705–13. 27920706 10.1159/000452403 PMC5126596

[4] Gonzalez RS , Salaria SN , Bohannon CD , *et al*. PD‐1 inhibitor gastroenterocolitis: Case series and appraisal of 'immunomodulatory gastroenterocolitis’. Histopathology 2017; 70: 558–67. 28000302 10.1111/his.13118

[5] Onuki T , Morita E , Sakamoto N , *et al*. Severe upper gastrointestinal disorders in pembrolizumab‐treated non‐small cell lung cancer patient. Respirol Case Rep 2018; 6: e00334. 30065841 10.1002/rcr2.334 PMC5980602

[6] Yang J , Lagana SM , Saenger YM , Carvajal RD . Dual checkpoint inhibitor‐associated eosinophilic enteritis. J Immunother Cancer 2019; 7: 310. 31730503 10.1186/s40425-019-0743-5 PMC6858706

[7] Rapisuwon S , Izar B , Batenchuk C , *et al*. Exceptional response and multisystem autoimmune‐like toxicities associated with the same T cell clone in a patient with uveal melanoma treated with immune checkpoint inhibitors. J Immunother Cancer 2019; 7: 61. 30832716 10.1186/s40425-019-0533-0 PMC6399858

[8] Duval L , Habes S , Chatellier T , *et al*. Nivolumab‐induced celiac‐like enteropathy in patient with metastatic renal cell carcinoma: Case report and review of the literature. Clin Case Rep 2019; 7: 1689–93. 31534728 10.1002/ccr3.2342 PMC6745396

[9] Hayashi Y , Hosoe N , Takabayashi K , *et al*. Clinical, endoscopic, and pathological characteristics of immune checkpoint inhibitor‐induced gastroenterocolitis. Dig Dis Sci 2020; 66: 2129–34. 32621258 10.1007/s10620-020-06441-w

[10] Irshaid L , Robert ME , Zhang X . Immune checkpoint inhibitor–induced upper gastrointestinal tract inflammation shows morphologic similarities to, but is immunologically distinct from, helicobacter pylori gastritis and celiac disease. Arch Pathol Lab Med 2021; 145: 191–200. 33501492 10.5858/arpa.2019-0700-OA

